# Pricing Constraint and the Complexity of IPO Timing in the Stock Market: A Dynamic Game Analysis

**DOI:** 10.3390/e22050546

**Published:** 2020-05-13

**Authors:** Zhiqiang Hu, Yuan Hu, Yushan Jiang, Zhen Peng

**Affiliations:** 1 Economics and Management School, Wuhan University, Wuhan 430072, China; huzq@whu.edu.cn (Z.H.); yuanhuems@whu.edu.cn (Y.H.); gai.peng@whu.edu.cn (Y.J.); 2 School of Business, Hubei University, Wuhan 430062, China

**Keywords:** pricing constraint, IPO timing, dynamic game model, real option, complexity of IPOs

## Abstract

The timing of an initial public offering (IPO) is a complex dynamic game in the stock market. Based on a dynamic game model with the real option, this paper investigates the relationship between pricing constraint and the complexity of IPO timing in the stock market, and further discusses its mechanism. The model shows that the IPO pricing constraint reduced the exercise value of the real option of IPO timing, thus restricting the enterprise’s independent timing and promoting an earlier listing. The IPO price limit has a stronger effect on high-trait enterprises, such as technology enterprises. Lowering the upper limit of the pricing constraint increases the probability that enterprises are bound by this restriction during IPO. A high discount cost and stock-market volatility are also reasons for early listing. This paper suggests a theoretical explanation for the mechanism of the pricing constraint on IPO timing in the complex market environment, which is an extension of IPO timing theory, itself an interpretation of the IPO behavior of Chinese enterprises. These findings provide new insights in understanding the complexity of IPOs in relation to the Chinese stock market.

## 1. Introduction

The discussion of the initial public offering (IPO) timing mechanism is one of the current hot topics in the field of corporate finance and financial market. Existing discussions on the IPO timing of enterprises are mostly derived from the research on IPO anomalies [1], which explained the internal logic of IPO timing from the perspective of enterprises themselves and market volatility [2]. The financial markets are complex systems [3]; however, the timing of an IPO is a complex game, which is influenced by many factors, both at the market level and macro level, and the game in IPO timing constitutes one part of the complexity in the stock market. When discussing the factors of IPO timing at the market level, the existing literature often talks about the role of information asymmetry and investor sentiment. There is little literature on the possible market regulation of IPOs, especially on the effect of price constraints on IPO timing.

Unlike overseas IPO markets, the issuance of new shares in the Chinese stock market is still at an emerging stage, and the corresponding issuance system is being gradually built and improved. In order to maintain the order of the issuing market and alleviate the “three highs” phenomenon (high issue price, high price-to-earnings (P/E) ratio, and high issue financing quota) in the actual issuance of new shares, the China Securities Regulatory Commission launched a series of reforms on the pricing of new shares, including setting the P/E ratio control on the pricing of new shares. In view of this, we studied the pricing constraint in IPO timing, which is a valuable expansion of the IPO timing theory, and complements the lack of attention given to market regulation in the previous literature.

On the other hand, the financing for technology companies is also a hot topic in the field of corporate finance. With the establishment of the China STAR Market (the Sci-Tech innovation board of the Shanghai Stock Exchange (SSESTAR), was established in the Shanghai Stock Market on the November 5, 2018. It is a new board independent of the existing main board market. Its main purpose is to pilot IPO registration reform and provide support for technological innovation of enterprises), many Chinese enterprises will choose to make an IPO in this market in future. Compared with the past, the SSESTAR Market has abolished the regulation of the P/E ratio and relieved the IPO price limit. This paper provides a theoretical foundation for that policy design. Specifically, compared with other enterprises, the pricing constraint has a stronger constraint on the independent IPO timing of technology IPOs. The pricing constraint will encourage them to go public earlier, which may further lead to insufficient financing, and thus weaken the IPO’s attraction of the domestic market for enterprises in the long term. Therefore, this research suggested that the release of restrictions on the IPO pricing may help to encourage companies to make an IPO in the Chinese stock market, enhancing the positive impact of capital market financing on the development of China’s innovation economy.

In this paper, we studied the influence of the pricing constraint on IPO timing based on a dynamic game model with the real option. The timing of an IPO is a complex multi-factor dynamic game, which is not only influenced by the enterprises themselves and the stock market fluctuations but also market regulation policy. The innovations of this research are as follows: first, it is research on the pricing constraint in IPO timing. The previous literature focused on the analysis of enterprise and market factors but overlooked the regulatory facts. This paper analyzes the influence of market regulation, especially for technology enterprises, and this is an expansion of the IPO timing theory. Second, our model incorporates market control factors into the dynamic mechanism of IPO timing, making the modeling more complex. By the introduction of multiple influencing factors and the real option, the model is much closer to the actual situation of IPO timing in the Chinese market. Third, we investigate the impact of the pricing constraint on technology enterprises in IPO timing. Our results prove that the price constraint can significantly advance the IPO timing of technology enterprises, and the resulting insufficiency of financing may be one of the reasons why Chinese enterprises seek to make an IPO in the overseas market, which provides new insights for understanding the complexity of the IPOs in relation to the Chinese stock market.

A breakdown of the paper is structured as follows: Section 2 is the literature review, where we review those studies related to IPO timing and the modeling of IPOs. Section 3 illustrates the parameters, settings, and important mechanisms of our model. Section 4 contains the derivation of the IPO timing dynamic game model. We first studied the IPO market timing equilibrium with no constraints, and then extended to an analysis of price limit constraints. Section 5 is the conclusion, in which we review the conclusions of this paper and indicate possible research directions for future study.

## 2. Literature Review

### 2.1. IPO Timing

The previous literature focused on the following two aspects to explore the motivation and results of IPO timing. On the one hand, the existence of information asymmetry in the stock market may lead to the distortion of IPO pricing, thus leading enterprises to choose the time of high financing to go public, “in light of the time conditions”. To increase the amount of listed capital, the enterprises to be listed tend to raise capital by listing when the market values the new shares highly, and delay the execution when the market values the new shares poorly. This theoretical hypothesis is supported by the empirical data.

Ibbotson et al. [4] and Korajczyk [5] found through empirical studies on the data of IPO enterprises in the market that enterprises would choose to go public when they could provide more accurate pricing of new shares, and that there was a positive correlation between IPO earnings and the number of new shares issued in subsequent markets. Lucas and McDonald [6] further believed that the existence of information asymmetry would result in the IPO price of enterprises being overestimated by the market. Then, the issuing of new shares when the IPO price was overestimated and suspension of the issuance when the IPO price was underestimated, verifying the motivation of enterprises in actively choosing the listing time.

On the other hand, some researchers believed that investor sentiment in the stock market is also a potential factor influencing the IPO timing of enterprises. The discussion in this respect comes from the expansion of the theory of investor sentiment. When the investor sentiment in the stock market keeps rising, it is accompanied by a general overvaluation of stock prices. At this point, the enterprises to be listed will issue IPO shares at a high price by virtue of high investor sentiment [7,8,9]. In addition, in the new issue, a large number of optimistic investors tend to gather in the primary market, and the pursuit of optimistic investors for a new issue will lead to a herd effect in the market. Therefore, when there are a large number of optimistic investors in the market, the overall optimistic market sentiment will push up the IPO price and underpricing rate of the IPO companies, stimulate the issuance of new shares, and then induce an IPO wave in the industry.

### 2.2. IPO Game Modeling

For the modeling of the IPO timing mechanism, the theoretical modeling of information asymmetry is the first step. Chemmanur and Fulghieri [10] proposed a two-phase IPO game decision-making model. They suggested that enterprises would waver between an external IPO financing or selling part of the equity. Their model concluded that the longer an enterprise has been operating, the more information it has publicly disclosed to the stock market, and the more likely it is to IPO. Therefore, the subsequent literatures discussed the information asymmetry in IPO from the information spillover effect.

Hoffmann-Burchardi [11] introduced the information spillover effect into the IPO timing model, believing that the existing IPO events can provide market investors with information about the industry and macro economy of the enterprise, thus affecting the IPO pricing of those subsequent IPO enterprises. The model shows that enterprises will choose to delay the IPO timing until the market obtains information from the IPOs of enterprises so as to price the enterprises that are about to go public more accurately. Alti [12], whose model is similar to Hoffmann-Burchardi [11], analyzed the information spillover effect of IPO events from the perspective of cost and benefit. The model analysis suggested that the cost of listing is caused by the existence of informed traders, which leads to adverse pricing in the market. However, the occurrence of IPO events in the past will reduce the degree of information asymmetry in the market and reduce the listing cost of subsequent listed companies.

Colak and Gunay [13] analyzed the macroeconomic factors in the spillover of IPO information. They got two Bayesian–Nash equilibriums for IPO timing. The first hypothesis is that the decision-making is independent, the quality of the enterprises listed in the early stage is often poor, and the enterprises with good quality will wait strategically and go public only after receiving the signal of the improvement of the macro economy. The second is that enterprises’ market timing will affect each other, and thus the probability of high-quality enterprises listing after the IPO of low-quality enterprises is higher in this situation.

In the theoretical modeling of IPO timing of investor sentiment, the model of Pastor and Veronesi [14] emphasizes the close relationship between IPO timing and investor sentiment. By introducing changes in investor sentiment towards the market as a whole, they assumed that companies had US-style call options because they could flexibly choose the timing of their listings and could obtain excess returns by exercising the options, at the cost of forgoing the cost of investment if the market sentiment continued to deteriorate. The model indicates that, when the expected market return rate falls and the future profit margin and operational risk of enterprises rise, the probability of enterprises choosing to make an IPO increases.

After the extension of the modeling terms to the multi-period game model, scholars represented by Spielgel and Tookes [15] built more complex multi-period game models of IPO timing and incorporated the influences of technological innovation and product market competition into the IPO timing of enterprises. The results of the model analysis demonstrate that the time an enterprise chooses to go public is related to the degree of technological innovation, and the market share owned by the enterprise also affects the timing of the IPO.

Compared with the above model research, Aghamollay and Guttmanz [16] attempted to evaluate an enterprise’s internal value (that is, the enterprise characteristics), information asymmetry, and investor sentiment, to consider the main body of the enterprise in different periods of the IPO timing dynamic game. Their model provided a relatively complete framework for describing the intrinsic mechanism of IPO timing.

### 2.3. Literature Comment

There are many studies on the mechanism of IPO timing; however, the mechanism of IPO timing discussed in those literatures is incomplete. Between January 2005 and December 2008, the China Securities Regulatory Commission (CSRC) imposed a P/E ratio of 30 times on domestic IPOs to maintain market stability. Since the resumption of IPO issuance in June 2014, the CSRC has introduced a series of reforms on IPO pricing, including setting the most critical constraint on IPO pricing—the P/E ratio of 23 times. However, there are not many studies on the IPO pricing constraints, and the analysis of IPO pricing constraints and the IPO timing mechanism are relatively rarely discussed. This paper adds to the growing literature on the complexity in the stock market by including the investigation of IPO pricing constraints in the study of the IPO timing mechanism.

## 3. Model Specification

### 3.1. Parameters

The trait factor $\psi_{i}$, which reflects the business behavior, financial characteristics, technology research, and development of the enterprise, represents the intrinsic value of the enterprise. Before *t* = 1, each company can only observe their own trait factors, which are independent of each other. Companies are risk-neutral individuals and are bound to go public at some point in the period mentioned above. The enterprise discloses its idiosyncratic trait factor $\psi_{i}$ when it goes public, and this information is true without cost.

The market factor $c_{t}$ represents the information asymmetry, investor sentiment, and rational expectation adjustment in the stock market. We set the factor $c_{t}$ to follow the random first order autoregressive process of mean reversion, the AR (1) process [17,18]. This process is not only related to the adjustment of investors to rational expectations [19,20], but also related to investor sentiment and market information asymmetry. The specific settings of the AR (1) process are shown in Equation (1).

(1) $$c_{t}=\lambda c_{t-1}+\varepsilon_{t}$$

where $c_{t}$ is the value of the common factor in the period. $c_{1}$ is unknown in t=1, unless there is at least one IPO in the market, then the rest can observe $c_{1}$ at the end of this period.

The discounted cost r represents a series of potential costs, such as market share, revenue decline, and debt increase, that would be lost if the company delayed the IPO under other conditions.

Last, enterprises are advance homogeneous; that is, the trait factors of enterprises have the same idiosyncratic distribution. They also face the same discount rate r, and common factor $c_{t}$ effects in the same way.

All parameters included in this model are described as shown in Table 1.

### 3.2. Assumptions

There are N (N≥2) companies to be listed. The timing of the IPO is a three-period decision (*t* ∈ {0,1,2,3}). The valuation of the IPO depends on the combined effect of three factors: enterprise trait factor $\psi_{i}$, the factor representing the characteristics of the industry and even the market volatility $c_{t}$ and the discounted cost r.

When an enterprise conducts an IPO at any time *t*, the expected utility obtained from its listing (that is, the valuation level) is determined by Equation (2).

(2) $$u_{i,\eta }=\frac{E\left(\psi_{i}+c_{\eta }|\omega_{\eta }\right)}{{\left(1+r\right)}^{\eta -1}}$$

The IPO timing process in this model is shown below.

*t* = 0, enterprises can only observe their own characteristics.

*t* = 1, all the companies decide whether to make an IPO at the same time. If at least one company is listed in *t* = 1, then the IPO company will obtain its market valuation, and common factor $c_{1}$ is known for all companies.

*t* = 2, all the enterprises not listed in phase *t* = 1 will decide whether to make an IPO at this time. If at least one company is listed in phase *t* = 2, the enterprises carrying out the IPO will also obtain their market valuation, and the common factor $c_{2}$ will be observed by all enterprises.

*t* = 3, all companies not listed in *t* = 1 and *t* = 2 will be listed in *t* = 3.

The IPO behavior of enterprises will affect each other, and the IPO events of the previous period in the market will affect the IPO decisions of the next period.

### 3.3. The Real Option Method

Referring to the research of Myers [21] and Busaba et al. [22] on the real option method in valuation, this paper introduces the options for enterprises to choose IPO timing. The IPO timing option is defined as the right of enterprises to choose IPO by observing the common factors of the previous period.

The model defines the expected utility of an enterprise (hereinafter referred to as a non-strategic enterprise) that does not execute the above strategic IPO timing and always chooses to list in *t* = 2 (hereinafter referred to as a non-strategic enterprise) as $NS\left(\varphi_{i}\right)$. The expected utility of an enterprise (hereinafter referred to as a strategic enterprise) that is not listed at *t* = 1 but at the time of a strategic IPO at *t* = 2 is defined as $S\left(\varphi_{i}\right)$. Therefore, the IPO timing real option $V_{t}\left(\varphi_{i}\right)$ is the difference between the expected utility of an IPO of strategic enterprises and non-strategic enterprises, as shown in Equations (3)–(5).

(3) $$NS\left(\psi_{i}\right)=E\left[\frac{\psi_{i}}{1+r}\right]=\frac{\psi_{i}}{1+r}$$

(4) $$S\left(\psi_{i}\right)=Pr\left(c_{2}<c_{2}^{*}\left(\psi_{i}\right)\right)E\left[\frac{\psi_{i}+c_{3}}{{\left(1+r\right)}^{2}}\right]+\left(1-Pr\left(c_{2}<c_{2}^{*}\left(\psi_{i}\right)\right)\right)E\left[\frac{\psi_{i}+c_{2}}{1+r}\right]$$

(5) $$V_{t}\left(\psi_{i}\right)\equiv S\left(\psi_{i}\right)-NS\left(\psi_{i}\right)=\int_{-\infty }^{c_{2}^{*}\left(\psi_{i}\right)}\left(\frac{\psi_{i}+\lambda^{2}c_{1}}{{\left(1+r\right)}^{2}}-\frac{\psi_{i}+\lambda c_{1}}{1+r}\right)f\left(c_{t}\right)dc_{t}$$

## 4. The IPO Timing Model

Compared with the discussion of market factors in the previous literature, this model places emphasis on the analysis of the regulatory factors of the stock market. Specifically, on the basis of Aghamollay and Guttmanz [16], this paper introduces the setting of IPO pricing constraints to explore the impact mechanism of pricing control measures on the enterprise’s IPO decision.

Enterprises maximize the expected utility of IPO (valuation). For any enterprise i, the IPO will be conducted at *t* = 1 only when its trait factors $\psi_{i}$ are higher than the critical trait factors $\psi_{i}^{*}$ and the expected utility of the current IPO is higher. Similarly, at *t* = 2, if an existing enterprise j is listed at *t* = 1, the enterprise i≠j will make an IPO at the stage if, and only if, its characteristic factors are above the critical value $\psi_{2}^{*}$. At the same time, when there is at least one IPO in *t* = 1, the enterprise i may delay the IPO to observe the specific information of the common factors $c_{1}$ at the end of *t* = 1. If the common factor is low enough at this time, considering that the volatility of the common factor follows the mean reversion process AR (1), its value at *t* = 3 will be greater than that at *t* = 2. In that case, the enterprise can delay its IPO to obtain the real option income generated by it.

Therefore, enterprise i will weigh the real option obtained by delaying the IPO at *t* = 1 against the discounted costs it faces, to finally determine the IPO period. After this section, the critical equilibrium of the dynamic game of IPO market timing will be solved. First, the equilibrium of the IPO market timing of the enterprise i in *t* = 2 will be discussed, and then the convergence in *t* = 1 will be discussed.

### 4.1. The Unconstrained Model

According to the setting, if there is no IPO in *t* = 1, the dominant choice of any enterprise i at this time is to make an IPO in *t* = 2.

If there is at least one IPO in stage *t* = 1, for the enterprises i that have not yet had an IPO in stage *t* = 1, if, and only if, their expected utility in a stage *t* = 3 and stage *t* = 2 IPO is equal, the enterprises will remain neutral to IPO in *t* = 2 (strategic delaying); otherwise, the enterprise should make an IPO in the current period of *t* = 2, as shown in Equation (6).

(6) $$\frac{\psi_{i}+E\left(c_{2}|\omega_{1}\right)}{1+r}=\frac{\psi_{i}+E\left(c_{3}|\omega_{1}\right)}{{\left(1+r\right)}^{2}}$$

The critical condition of IPO timing at *t* = 2 is shown in Equation (7).

(7) $$\psi_{i}\geq \psi_{2}^{*}\left(c_{1}\right)=-c_{1}\left(1+r-\lambda \right)\left(\frac{\lambda }{r}\right)$$

Furthermore, if the enterprise i makes an IPO in *t* = 1, its expected benefits are fixed as $\psi_{i}+E\left(c_{1}\right)=\psi_{i}$. If the enterprise delays the IPO at *t* = 1, its expected utility is related to the IPO situation in the stock market during the same period.

To be specific: (1) when there is no IPO in the stock market of *t* = 1, the enterprise will be listed in *t* = 2, the expected utility is $\frac{E\left(\psi_{i}+c_{2}\right)}{1+r}=\frac{\psi_{i}}{1+r}$. (2) If there is at least one IPO in the stock market of *t* = 1 and the characteristics of the enterprise are higher than the critical condition, $\psi_{i}>\psi_{i}^{*}\left(c_{2}\right)$, the enterprise will make an IPO in *t* = 2, and the expected utility is $\frac{E\left(\psi_{i+}c_{2}|\psi_{i}>\psi_{i}^{*}\left(c_{2}\right)\right)}{1+r}$. (3) IPOs happened at *t* = 3 to obtain the real option proceeds from delay; in this case, the IPO expected utility is $\frac{E\left(\psi_{i}+c_{3}|\psi_{i}<\psi_{i}^{*}\left(c_{2}\right)\right)}{{\left(1+r\right)}^{2}}$. Thus, the expected utility of enterprise i obtained from the delay of the IPO at *t* = 1 is shown in Equation (8).

(8) $$Pr\left(NI_{i\neq j}^{1}\right)\left(\frac{\psi_{i}}{1+r}\right)+\left(1-Pr\left(NI_{i\neq j}^{1}\right)\right)\left(\begin{array}{c} Pr\left(I_{i\neq j}^{1}\right)E\left[payoff\;at\;t=2|\psi_{i},I_{i}^{2}\right]\\ +Pr\left(NI_{i\neq j}^{1}\right)E\left[payoff\;at\;t=3|\psi_{i},NI_{i}^{2}\right] \end{array}\right)$$

Because the trait factor $\psi_{i}$ is independent of each enterprise, the probability of an IPO is the same for any enterprise i. By introducing the cumulative density function correlation of the above variable, the critical condition of IPO in *t* = 1 is shown in Equation (9), the derivation is shown in Appendix A.

(9) $$\begin{array}{l} \psi_{1}^{*}\\ ={\left[G\left(\psi_{1}^{*}\right)\right]}^{N-1}\left(\frac{\psi_{1}^{*}}{1+r}\right)\\ +(1\\ -{\left[G\left(\psi_{1}^{*}\right)\right]}^{N-1})\left(\begin{array}{c} F\left(\frac{\psi_{1}^{*}}{\left(1+r-\lambda \right)\left(\frac{\lambda }{r}\right)}\right)\frac{\psi_{1}^{*}}{1+r}+\frac{1}{1+r}\lambda \sigma^{2}f\left(-\frac{\psi_{1}^{*}}{\left(1+r-\lambda \right)\left(\frac{\lambda }{r}\right)}\right)\\ +\left(1-F\left(\;\frac{\psi_{1}^{*}}{\left(1+r-\lambda \right)\left(\frac{\lambda }{r}\right)}\right)\right)\frac{\psi_{1}^{*}}{1+r}-\frac{1}{{\left(1+r\right)}^{2}}\lambda^{2}\sigma^{2}f\left(-\frac{\psi_{1}^{*}}{\left(1+r-\lambda \right)\left(\frac{\lambda }{r}\right)}\right) \end{array}\right) \end{array}$$

Comparing the critical conditions of the IPO timing in *t* = 2 and *t* = 1 without pricing constraints, we can give Proposition 1 as below.

**Proposition** **1.** *The higher the characteristics of an enterprise, the earlier the optimal listing time.*

### 4.2. IPO Timing with a Pricing Constraint

In order to limit the three high issues in the process of new issues, the CSRC has introduced a variety of measures to limit the IPO issue price of enterprises, and then to restrict the amount of financing obtained by enterprises. When there is an issue pricing constraint in new issue pricing, this paper sets the expected utility of enterprises to make a new issue at the upper limit of pricing. For any enterprise making an IPO, the expected utility function for the enterprises’ IPO is shown in Equation (10).

(10) $$u_{i,\eta }=\frac{min\left\{E\left(\psi_{i}+c_{\eta }|\omega_{\eta }\right),P^{*}\right\}}{{\left(1+r\right)}^{\eta -1}}$$

When enterprise *i* is unable to observe the information of market common factors, its decision is similar to that of unconstrained pricing. On the contrary, the utility of enterprises in delaying an IPO to *t* = 2 is reduced, so all enterprises will choose to go public at *t* = 1.

If enterprise *i* can observe the market common factors of the period and the market common factors of the period is greater than zero or equals to zero ($c_{1}\geq 0$), due to the mean recovery feature of the market common factors, the enterprise cannot obtain the real option by delaying. Therefore, all unlisted companies in *t* = 1 will be listed as soon as possible in *t* = 2.

If the common factor, observed in *t* = 1, is $c_{1}\leq 0$, for all companies not making an IPO on *t* = 1 but at *t* = 2, the expected utility for the IPO is shown in Equation (11).

(11) $$\frac{min\left\{E\left(\psi_{i}+c_{2}|c_{1}\right),P^{*}\right\}}{1+r}=\frac{min\left\{\psi_{i}+\lambda c_{1},P^{*}\right\}}{1+r}$$

When the IPO is postponed to *t* = 3, the expected utility for the IPO is shown in Equation (12).

(12) $$\frac{min\left\{E\left(\psi_{i}+c_{3}|c_{1}\right),P^{*}\right\}}{{1+r}^{2}}=\frac{min\left\{\psi_{i}+\lambda c_{1},P^{*}\right\}}{{1+r}^{2}}$$

where $P^{*}$ is the ceiling of the IPO’s expected utility. Due to the existence of the maximum price ceiling in IPO pricing, enterprises will face the following three situations: (1) in *t* = 2 and *t* = 3, they will be subject to the issue pricing constraints, and all of them will be priced according to the price ceiling to obtain the expected utility. (2) They are not subject to the issue pricing constraint in term *t* = 2, whereas they are subject to the issue pricing constraint in term *t* = 3, and this is issued according to the upper limit of the pricing. (3) The IPO in *t* = 2 and *t* = 3 will not be subject to the issue pricing.

#### 4.2.1. Critical Conditions

Referring to the paradigm of this basic model, this section first analyzes the IPO timing equilibrium of *t* = 2, and then discusses the IPO timing equilibrium of *t* = 1. In addition, the common market factors of *t* = 1 in this part should be $c_{1}\leq 0$ (according to the above analysis, if $c_{1}\geq 0$, the enterprise i, should choose to go public in *t* = 2 as early as possible).

The critical equilibrium of IPO timing at *t* = 2 is shown below.

(1) Pricing constrained in both *t* = 2 and *t* = 3

In this case, the trait factor $\psi_{i}$ of enterprise i is in the range shown in Equation (13).

(13) $$\psi_{i}+\lambda^{2}c_{1}>\psi_{i}+\lambda c_{1}>P^{*}$$

The trait factor $\psi_{i}>P^{*}-\lambda c_{1}$, the expected utility of IPOs in *t* = 2 is $\frac{P^{*}}{{\left(1+r\right)}^{2}}$, and the probability of an IPO is $F\left(\frac{\psi_{i}-P^{*}}{\lambda }\right)$. Due to the pricing constraint, enterprise i could not obtain the proceeds from the exercise of IPO timing; therefore, it must make an IPO as soon as possible in *t* = 2.

(2) Pricing unconstrained in *t* = 2 but pricing constrained in *t* = 3

In this case, the trait factor $\psi_{i}$ of the enterprise is in the range shown in Equation (14).

(14) $$\psi_{i}+\lambda^{2}c_{1}>P^{*}>\psi_{i}+\lambda c_{1}$$

The trait factor $P^{*}-\lambda c_{1}>\psi_{i}>P^{*}-\lambda^{2}c_{1}$, the expected utility of IPOs in *t* = 2 is $\frac{\psi_{i}+\lambda c_{1}}{1+r}$, and the probability of an IPO is $F\left(\frac{P^{*}-\psi_{i}}{\lambda^{2}}\right)+F\left(\frac{P^{*}-\psi_{i}}{\lambda }\right)-1$. After delay to *t* = 3, the IPO’s expected utility is $\frac{P^{*}}{{\left(1+r\right)}^{2}}$. The critical condition of IPO in *t* = 2 is shown in Equation (15).

(15) $$\psi_{i}=\psi_{2}^{*}\left(c_{1}\right)=\frac{P^{*}-\lambda \left(1+r\right)c_{1}}{1+r}$$

Thus, only if the trait-factor $\psi_{i}\geq \psi_{2}^{*}\left(c_{1}\right)$, the enterprise i chooses to make an IPO in *t* = 2.

(3) Pricing unconstrained in both *t* = 2 and *t* = 3

In this case, the trait factor $\psi_{i}$ of the enterprise is in the range shown in Equation (16).

(16) $$P^{*}>\psi_{i}+\lambda^{2}c_{1}>\psi_{i}+\lambda c_{1}$$

The trait factor follows $\psi_{i}<P^{*}-\lambda^{2}c_{1}$, and the probability is $F\left(\frac{P^{*}-\psi_{i}}{\lambda^{2}}\right)$. We skip the analysis here, as it is consistent with the unconstrained IPO timing.

The critical equilibrium of IPO timing at *t* = 1 is shown below.

(1) Pricing constrained in both *t* = 2 and *t* = 3

Whether there is an IPO in *t* = 1 or not, the IPO’s expected utility obtained by enterprise i in *t* = 2 is $\frac{P^{*}}{1+r}$, lower than the expected utility $P^{*}$ at *t* = 1. As a result, the enterprise i will choose to make an IPO at *t* = 1 rather than delaying.

(2) Pricing unconstrained in *t* = 2 but pricing constrained in *t* = 3

If there is no IPO in the market of *t* = 1, then the enterprise i will be listed at *t* = 2, and the expected utility is $\frac{P^{*}}{1+r}$; if there is at least one IPO in *t* = 1, when the trait factor is higher than the critical condition of *t* = 2, the expected utility of the IPO in *t* = 2 is $\frac{E\left(\psi_{i+}c_{2}|\psi_{i}>\psi_{i}^{*}\left(c_{2}\right)\right)}{1+r}$; otherwise, the IPO will be postponed to *t* = 3 and the expected utility is $\frac{P^{*}}{{\left(1+r\right)}^{2}}$.

The expected utility obtained by the enterprise in the IPO of *t* = 1 is $P^{*}$. The critical condition for the IPO at *t* = 1 is the trait factor of $\psi_{1}^{*}$, which equals the expected utility $P^{*}$ at *t* = 1, as shown in Equation (17), the derivation is shown in Appendix A.

(17) $$\begin{array}{l} P^{*}\\ ={\left[G\left(\psi_{1}^{*}\right)\right]}^{N-1}\left(\frac{P^{*}}{1+r}\right)\\ +\left(1-{\left[G\left(\psi_{1}^{*}\right)\right]}^{N-1}\right)\left(\begin{array}{c} F\left(\;\frac{\left(1+r\right)\psi_{1}^{*}-P^{*}}{\lambda \left(1+r\right)}\right)\frac{\psi_{1}^{*}}{1+r}+\frac{\lambda }{1+r}\sigma^{2}f\left(\frac{P^{*}-\left(1+r\right)\psi_{1}^{*}}{\lambda \left(1+r\right)}\right)\\ +\left(1-F\left(\;\frac{\left(1+r\right)\psi_{1}^{*}-P^{*}}{\lambda \left(1+r\right)}\right)\right)\frac{P^{*}}{{\left(1+r\right)}^{2}} \end{array}\right) \end{array}$$

#### 4.2.2. Comparative Static Analysis

(1) Pricing constraint in both *t* = 2 and *t* = 3

In this case, all enterprises will choose to make an IPO as soon as possible in phase *t* = 1, so there is no comparative static analysis of critical conditions in *t* = 2.

(2) Pricing unconstrained in *t* = 2 but pricing constrained in *t* = 3

The critical condition of the IPO in *t* = 2 is shown in Equation (18).

(18) $$\psi_{2}^{*}\left(c_{1}\right)\equiv \frac{P^{*}-\lambda \left(1+r\right)c_{1}}{1+r}$$

With the other conditions unchanged, in this section, we discuss the influence of the changes of the following three factors on the critical conditions of *t* = 2 IP: (a) the discount rate r; (b) the common factors recovery rate λ; (c) the upper limit $P^{*}$ of IPO utility with a pricing constraint, as shown in Equations (19)–(21).

(19) $$\frac{\partial }{\partial \lambda }\psi_{2}^{*}\left(c_{1}\right)=-\frac{P^{*}}{{1+r}^{2}}<0$$

(20) $$\frac{\partial }{\partial \lambda }\psi_{2}^{*}\left(c_{1}\right)=-c_{1}>0$$

(21) $$\frac{\partial }{\partial P^{*}}\psi_{2}^{*}\left(c_{1}\right)=\frac{1}{1+r}>0$$

These calculated results show that with the increase in discount cost, the critical condition of an IPO will decrease, which is consistent with the conclusion when there is no pricing constraint, and this also reflects the early listing tendency of the enterprise itself. There is only one path for the influence of the common factor mean recovery rate λ on the critical condition of an IPO in *t* = 2. The increase in λ indicates that the accuracy of predicting the future stock market conditions will be improved, which will increase the extra profit that companies can obtain from delaying their IPO. The raising of the upper limit of pricing constraint $P^{*}$ will raise the critical condition of the IPO decision, if the IPO pricing limit is set in the market, the increase in the IPO pricing limit can relax the restrictions on the IPO timing, and the enterprises may gain additional expected utility from this IPO delay.

On the influence on the critical condition of an IPO decision in *t* = 1, the discount rate is negative. As for the recovery speed of common factors, in this section, we deduce its influence by analyzing the real option and the critical condition of the timing of *t* = 2 IPOs. According to the model, the IPO real option is shown in Equation (22), the derivation is shown in Appendix B.

(22) $$V_{2}\left(\psi_{i}\right)=\int_{-\infty }^{c_{2}^{*}\left(\psi_{i}\right)}\left(\frac{P^{*}}{{\left(1+r\right)}^{2}}-\frac{\psi_{i}+\lambda c_{1}}{1+r}\right)f\left(c_{1}\right)dc_{1}$$

Then, we solve for the partial derivatives, as shown in Equation (23).

(23) $$\frac{\partial V_{2}\left(\psi_{i}\right)}{\partial \lambda }=\int_{-\infty }^{c_{2}^{*}\left(\psi_{i}\right)}\left(\frac{1}{1+r}\right)f\left(c_{1}\right)dc_{1}>0$$

As the common factor mean recovery rate  λ grows, enterprises can take advantage of the real option by delaying their IPOs. Thus, the higher the recovery speed λ, the higher the critical condition of an IPO in *t* = 1, for the partial differential of pricing upper limit $P^{*}$, as shown in Equation (24).

(24) $$\frac{\partial }{\partial P^{*}}V_{2}\left(\psi_{i}\right)=\frac{1}{{\left(1+r\right)}^{2}}F\left(\frac{P^{*}}{1+r}-\psi_{i}\right)+\left(\frac{\left(1-\lambda \right)P^{*}}{1+r}-\left(1-\lambda \right)\psi_{i}\right)f\left(\frac{P^{*}}{1+r}-\psi_{i}\right)$$

The calculations suggest that, although there may be a critical value of $P^{*}$, the further derivation shows that the reduction in the upper limit of IPO pricing $P^{*}$ will reduce the value of the IPO timing option, and reduce the critical condition of IPO timing of both *t* = 1 and *t* = 2, thus leading to the early listing of enterprises.

### 4.3. The Effects of the Pricing Constraint

Facing the IPO pricing constraint and regulation in the stock market, if the expected price of an enterprise exceeds that ceiling, it has to re-price according to the ceiling and obtain the corresponding expected utility $P^{*}$. Enterprises will face different situations of the pricing constraints according to their own trait factors. This section will expand in two aspects: the restricted probability and IPO timing critical condition.

#### 4.3.1. Restricted IPO Probability

Keeping the trait factors of enterprises unchanged, when the IPO price ceiling $P^{*}$ is lowered, the probability of IPO $F\left(\frac{\psi_{i}-P^{*}}{\lambda }\right)$ grows, the IPO probability $\left[F\left(\frac{P^{*}-\psi_{i}}{\lambda^{2}}\right)+F\left(\frac{P^{*}-\psi_{i}}{\lambda }\right)-1\right]$ goes down, and the probability $F\left(\frac{P^{*}-\psi_{i}}{\lambda^{2}}\right)$ declines. On the other hand, if the price ceiling $P^{*}$ is fixed, our research shows that the higher the trait factors $\psi_{i}$, the higher the probability $F\left(\frac{\psi_{i}-P^{*}}{\lambda }\right)$; however, probability $\left[F\left(\frac{P^{*}-\psi_{i}}{\lambda^{2}}\right)+F\left(\frac{P^{*}-\psi_{i}}{\lambda }\right)-1\right]$ and probability $F\left(\frac{P^{*}-\psi_{i}}{\lambda^{2}}\right)$ decline.

#### 4.3.2. IPO Timing Critical Condition

After the implementation of price control on the new issue pricing, the enterprises are faced with different pricing constraints due to differences in the trait factors. Our model analysis showed that pricing constraint reduces the critical condition of IPO timing when *t* = 2. At this time, the pricing constraints lead to those enterprises with high trait factor can only price according to the price ceiling at *t* = 3, thus reducing the value of the real option obtained in the delayed IPO, so they are more inclined to go public at *t* = 2 in advance.

The above analysis leads to Propositions 2–4, as below.

**Proposition** **2.** *Lowering the ceiling on prices increases the probability that enterprises will be affected by IPO pricing constraints.*

**Proposition** **3.** *The price constraint has a higher probability of influencing the IPO of high trait factor enterprises.*

**Proposition** **4.** *The existence of a pricing constraint will reduce the critical condition of the IPO, which promotes enterprises going public in advance.*

### 4.4. Discussion

In this section, we discuss the conclusions with the previous studies. In previous literatures, the studies of enterprises’ IPO decision focused on the factor of enterprises’ intrinsic value [2,23], information asymmetry [11], and market investor sentiment [7,8,9] individually, but they failed to explain the complexity of IPOs. This paper integrates the three factors of enterprises’ intrinsic value, information asymmetry and market investor sentiment into the united theoretical model of enterprises’ IPO decision, and studies the complexity of the mechanisms.

The price behavior in the stock market is complex [24,25]. Our model provided new insights in understanding the relationship between IPOs and market trend. If the enterprise expects that the future stock market trend will be negative (meaning the common factor $c_{t}$ is negative in the future), whether there is IPO price control in the market or not, the dominant decision of the company is to go public as soon as possible at *t* = 1, which avoids delaying the listing and reduces the expected utility obtained by the IPO. The real option on this IPO timing will not be exercised yet.

If the enterprise expects the future stock market to be positive (the common factor $c_{t}$ will rise), then enterprises can exercise the real option of IPO timing to delay their IPO period to get a higher expected utility (valuation). However, when there is a pricing constraint, the value of the real option will decrease or even disappear, and the expected utility of IPOs is reduced and the IPO time has to be brought forward.

This paper can explain the complexity of IPOs [26,27,28]. First, it is expected that IPOs with a large financing scale will not happen in the Chinese stock market, as the issuance price constraint directly limits the financing quota of companies, and if the companies go public, they cannot obtain sufficient financing. Secondly, the valuation of technological innovation is also included in the trait factors. Due to the IPO pricing constraints, enterprises with high valuations of technological innovation will be restricted and listed earlier, which could further limit the amount of IPO capital and prompt them to stay private or change their IPO location.

**Figure 1:**
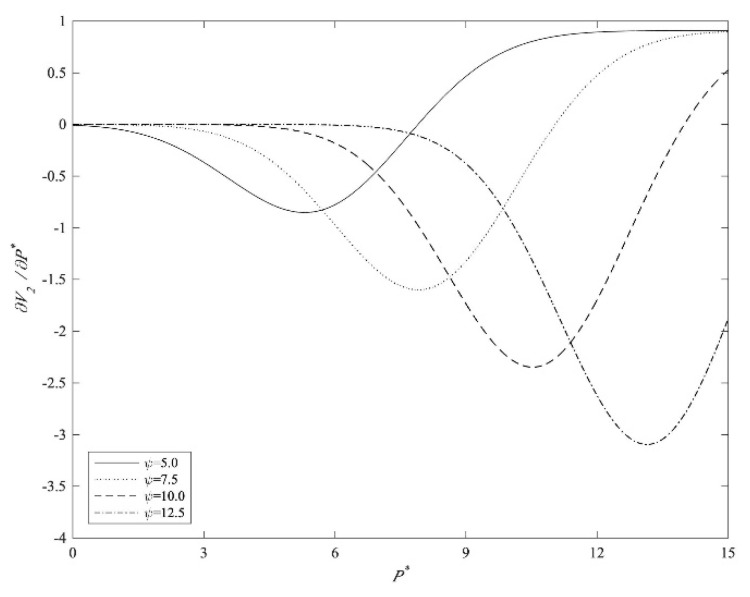
The effect of pricing constraints on the value of IPO real options. Note: The other parameters in the simulation are set as follows: r=0.05, λ=0.5, F(X) is the CDF of X ~ N (0,2). The result is robust when other values of the parameters are selected.

The core point of this paper is that pricing constraints will reduce the value of IPO real options, which can theoretically explain the Chinese companies’ IPO behavioral characteristics. Obviously, when an enterprise faces current IPO price constraints or future pricing constraints, listing as soon as possible to maximize its utility for the IPO should be the dominant choice for the enterprise. Figure 1 shows that the higher the characteristics of an enterprise (ψ), the greater the negative effect of pricing constraints ($P^{*}$) on the value of IPO real options (∂V2∂P*).

## 5. Conclusions

Based on the characteristics of the IPO limit in the Chinese stock market, this paper discussed the influence of pricing constraints on IPO timing, which is an extension of the IPO timing theory. In the process of IPO timing, enterprises not only need to comprehensively consider their own characteristics, the market fluctuations, and macroeconomic factors, but also must consider how the market regulation of the IPO will have a significant and different impact on their timing decision, which significantly increases the complexity of IPO decision modeling.

The IPO pricing constraint will short the waiting period of enterprises, that is, promote the enterprises going public in advance. This restriction has a more significant impact on those enterprises with high trait factors, such as technology enterprises. The early IPO of companies may hamper their ability to raise sufficient capital in the stock market and reduce their incentive to make an IPO on the Chinese stock market. Generally speaking, these findings provide new insights into understanding the complexity of IPOs in relation to the Chinese stock market.

However, this paper has the limitation that requires future study. The model assumes that the information disclosure of IPO enterprises is true and cost-free, whereas in the actual stock market, the cost of information disclosure, which is caused by information asymmetry and selective disclosure, exists. Therefore, clarifying the mechanism of information disclosure cost in the IPO market timing game should be the future development direction for the model.

## Figures and Tables

**Table 1:** Description of the parameters in the model.

Parameter	Definition of the Parameters
$\psi_{i}$	The trait factors of enterprise $i,\psi_{i}\epsilon \left(0,+\infty \right)$, i∈(1,N), N≥2.
$\psi_{t}^{*}$	The critical condition of whether the enterprise delays the IPO in period t. *t* = 1,2.
$c_{t}$	The common market factor in period *t*. $c_{t}\epsilon \left(-\infty ,+\infty \right)$, $c_{0}=0$, $c_{1}=\tilde{\varepsilon_{1}}$. $\varepsilon_{t}$ is the stochastic error term, $\varepsilon_{t}\sim N\left(0,\sigma^{2}\right)$.
$\omega_{t}$	The market environment variable at period *t*, which is closely related to the common market factor $c_{t}$ in the same period.
λ	The mean reversion rate of the common market factors, λ∈(0,1).
*r*	A collection of the discounted costs considered in an IPO. The discount rate stays the same for each period.
*t*	The decision time point of IPO timing in dynamic game model, *t* = 0, 1, 2, 3.
$u_{i,t}$	The expected utility of enterprises listing in period *t*, which reflects the market valuation of those enterprises.
F(·),f(·)	The cumulative distribution function and density function of the common market factor $c_{t}$.
G(·)	The cumulative distribution function of the trait factor $\psi_{i}$.
$Pr\left(NI_{i\neq j}^{t}\right)$	The probability of no IPO in the market at *t* period.
$Pr\left(I^{t}\right)$/$Pr\left(NI^{t}\right)$	The probability of an IPO for enterprise i at period *t*, the probability that enterprise i will not IPO.
$NS\left(\psi_{i}\right)$	The non-strategic enterprises. The expected utility of an IPO in a fixed period.
$S\left(\psi_{i}\right)$	The strategic enterprise. The expected utility of strategic IPO.
$V_{t}\left(\psi_{i}\right)$	The real option of IPO timing. The expected utility difference of an IPO between strategic and non-strategic enterprises.

## Appendix A

Derivation: The critical condition of IPO timing in *t* = 1 is shown in Equation (A1)

(A1) $$\begin{array}{c} Pr\left(NI_{i\neq j}^{1}\right)\left(\frac{\psi_{i}}{1+r}\right)+\left(1-Pr\left(NI_{i\neq j}^{1}\right)\right)\left(\begin{array}{c} Pr\left(I_{i\neq j}^{1}\right)E\left[payoff\;at\;t=2|\psi_{i},I_{i}^{2}\right]\\ +Pr\left(NI_{i\neq j}^{1}\right)E\left[payoff\;at\;t=3|\psi_{i},NI_{i}^{2}\right] \end{array}\right)\\ ={\left[G\left(\psi_{1}^{*}\right)\right]}^{N-1}\left(\frac{\psi_{1}^{*}}{1+r}\right)+\\ \left(1-{\left[G\left(\psi_{1}^{*}\right)\right]}^{N-1}\right)\left(\begin{array}{c} Pr\left(\psi_{i}>-c_{1}\left(1+r-\lambda \right)\left(\frac{\lambda }{r}\right)\right)E\left[\frac{\psi_{i}+c_{2}}{1+r}|\psi_{i}>c_{1}\left(1+r-\lambda \right)\left(\frac{\lambda }{r}\right)\right]\\ +\left(1-Pr\left(\psi_{i}>-c_{1}\left(1+r-\lambda \right)\left(\frac{\lambda }{r}\right)\right)\right)E\left[\frac{\psi_{i}+c_{3}}{{\left(1+r\right)}^{2}}|\psi_{i}\leq -c_{1}\left(1+r-\lambda \right)\left(\frac{\lambda }{r}\right)\right] \end{array}\right) \end{array}$$

At any point in time, the enterprise knows its own trait factors, as shown in Equation (A2).

(A2) $$Pr\left(\psi_{i}>-c_{1}\left(1+r-\lambda \right)\left(\frac{\lambda }{r}\right)\right)=Pr\left(c_{1}>-\frac{\psi_{i}}{\left(1+r-\lambda \right)\left(\frac{\lambda }{r}\right)}\right)=F\left(\frac{\psi_{i}}{\left(1+r-\lambda \right)\left(\frac{\lambda }{r}\right)}\right)$$

We can derive the Equation (A3).

(A3) $$\begin{array}{l} E\left[\frac{\psi_{i}+c_{2}}{1+r}|\psi_{i}>c_{1}\left(1+r-\lambda \right)\left(\frac{\lambda }{r}\right)\right]=E\left[\frac{\psi_{i}+c_{2}}{1+r}|c_{1}>\frac{\psi_{i}}{\left(1+r-\lambda \right)\left(\frac{\lambda }{r}\right)}\right]\\ \;\;\;\;\;\;\;\;\;\;\;\;\;\;\;\;\;\;\;\;=\frac{1}{F\left(\frac{\psi_{i}}{\left(1+r-\lambda \right)\left(\frac{\lambda }{r}\right)}\right)}\int_{-\frac{\psi_{i}}{\left(1+r-\lambda \right)\left(\frac{\lambda }{r}\right)}}^{\infty }\frac{\psi_{i}+E\left(c_{2}|c_{1}\right)}{1+r}f\left(c_{1}\right)dc_{1}\\ \;\;\;\;\;\;\;\;\;\;\;\;\;\;\;\;\;\;\;\;=\frac{\psi_{i}}{1+r}+\frac{1}{1+r}\frac{1}{F\left(\frac{\psi_{i}}{\left(1+r-\lambda \right)\left(\frac{\lambda }{r}\right)}\right)}\int_{-\frac{\psi_{i}}{\left(1+r-\lambda \right)\left(\frac{\lambda }{r}\right)}}^{\infty }\frac{E\left(c_{2}|c_{1}\right)}{1+r}f\left(c_{1}\right)dc_{1}\\ \;\;\;\;\;\;\;\;\;\;\;\;\;\;\;\;\;\;\;\;=\frac{\psi_{i}}{1+r}\\ \;\;\;\;\;\;\;\;\;\;\;\;\;\;\;\;\;\;\;\;+\frac{1}{1+r}\frac{1}{F\left(\frac{\psi_{i}}{\left(1+r-\lambda \right)\left(\frac{\lambda }{r}\right)}\right)}\left[\int_{-\frac{\psi_{i}}{\left(1+r-\lambda \right)\left(\frac{\lambda }{r}\right)}}^{\infty }\left(\lambda c_{1}+\varepsilon_{2}\right)f\left(c_{2}\right)dc_{2}\right]f\left(c_{1}\right)dc_{1}\\ \;\;\;\;\;\;\;\;\;\;\;\;\;\;\;\;\;\;\;\;=\frac{\psi_{i}}{1+r}+\frac{1}{1+r}\frac{1}{F\left(\frac{\psi_{i}}{\left(1+r-\lambda \right)\left(\frac{\lambda }{r}\right)}\right)}\int_{-\frac{\psi_{i}}{\left(1+r-\lambda \right)\left(\frac{\lambda }{r}\right)}}^{\infty }\lambda c_{1}f\left(c_{1}\right)dc_{1}\\ \;\;\;\;\;\;\;\;\;\;\;\;\;\;\;\;\;\;\;\;=\frac{\psi_{i}}{1+r}+\frac{\lambda }{1+r}E\left[c_{1}|c_{1}>\frac{\psi_{i}}{\left(1+r-\lambda \right)\left(\frac{\lambda }{r}\right)}\right] \end{array}$$

As $X\sim N\left(\mu_{X},\sigma^{2}\right)$, its expectation of truncated normal distribution is shown in Equation (A4).

(A4) $$E\left[X|X\in \left[a,b\right]\right]=\mu_{X}-\sigma^{2}\frac{f\left(a\right)-f\left(b\right)}{F\left(a\right)-F\left(b\right)}$$

If a≈−∞, $E\left[X|X<b\right]=\mu_{X}-\sigma^{2}\frac{f\left(b\right)}{F\left(b\right)}$ and if b≈∞, $E\left[X|X>a\right]=\mu_{X}+\sigma^{2}\frac{f\left(a\right)}{1-F\left(a\right)}$.

Thus, we have Equation (A5).

(A5) $$\begin{array}{c} E\left[\frac{\psi_{i}+c_{2}}{1+r}|\psi_{i}>c_{1}\left(1+r-\lambda \right)\left(\frac{\lambda }{r}\right)\right]=\frac{\psi_{i}}{1+r}+\frac{\lambda }{1+r}E\left[c_{1}|c_{1}>\frac{\psi_{i}}{\left(1+r-\lambda \right)\left(\frac{\lambda }{r}\right)}\right]\\ =\frac{\psi_{i}}{1+r}+\frac{\lambda }{1+r}\left(-\sigma^{2}\frac{-f\left(-\frac{\psi_{i}}{\left(1+r-\lambda \right)\left(\frac{\lambda }{r}\right)}\right)}{1-F\left(-\frac{\psi_{i}}{\left(1+r-\lambda \right)\left(\frac{\lambda }{r}\right)}\right)}\right) \end{array}$$

Similarly, we have Equation (A6).

(A6) $$\begin{array}{l} E\left[\frac{\psi_{i}+c_{3}}{1+r}|\psi_{i}\leq -c_{1}\left(1+r-\lambda \right)\left(\frac{\lambda }{r}\right)\right]\\ \;\;\;\;\;\;\;\;\;\;\;\;\;\;\;\;\;\;\;\;=\frac{\psi_{i}}{{\left(1+r\right)}^{2}}+\frac{\lambda^{2}}{{\left(1+r\right)}^{2}}E\left[c_{1}|c_{1}<-\frac{\psi_{i}}{\left(1+r-\lambda \right)\left(\frac{\lambda }{r}\right)}\right]\\ \;\;\;\;\;\;\;\;\;\;\;\;\;\;\;\;\;\;\;\;=\frac{\psi_{i}}{{\left(1+r\right)}^{2}}+\frac{\lambda^{2}}{{\left(1+r\right)}^{2}}\left(-\sigma^{2}\frac{-f\left(-\frac{\psi_{i}}{\left(1+r-\lambda \right)\left(\frac{\lambda }{r}\right)}\right)}{1-F\left(\frac{\psi_{i}}{\left(1+r-\lambda \right)\left(\frac{\lambda }{r}\right)}\right)}\right) \end{array}$$

The expected utility of enterprises in delaying their IPO at *t* = 1 is shown in Equation (A7).

(A7) $$\begin{array}{l} {\left[G\left(\psi_{1}^{*}\right)\right]}^{N-1}\left(\frac{\psi_{1}^{*}}{1+r}\right)+\left(1-{\left[G\left(\psi_{1}^{*}\right)\right]}^{N-1}\right)\\ {}*\left(\begin{array}{c} Pr\left(\psi_{i}>-c_{1}\left(1+r-\lambda \right)\left(\frac{\lambda }{r}\right)\right)E\left[\frac{\psi_{i}+c_{2}}{1+r}|\psi_{i}>c_{1}\left(1+r-\lambda \right)\left(\frac{\lambda }{r}\right)\right]\\ +\left(1-Pr\left(\psi_{i}>-c_{1}\left(1+r-\lambda \right)\left(\frac{\lambda }{r}\right)\right)\right)E\left[\frac{\psi_{i}+c_{3}}{{\left(1+r\right)}^{2}}|\psi_{i}\leq -c_{1}\left(1+r-\lambda \right)\left(\frac{\lambda }{r}\right)\right] \end{array}\right)={\left[G\left(\psi_{1}^{*}\right)\right]}^{N-1}\left(\frac{\psi_{1}^{*}}{1+r}\right)\\ +\left(1-{\left[G\left(\psi_{1}^{*}\right)\right]}^{N-1}\right)\\ {}*\left(\begin{array}{c} F\left(\frac{\psi_{1}^{*}}{\left(1+r-\lambda \right)\left(\frac{\lambda }{r}\right)}\right)\frac{\psi_{1}^{*}}{1+r}+\frac{\lambda \sigma^{2}}{1+r}f\left(-\frac{\psi_{1}^{*}}{\left(1+r-\lambda \right)\left(\frac{\lambda }{r}\right)}\right)\\ +\left(1-F\left(\frac{\psi_{1}^{*}}{\left(1+r-\lambda \right)\left(\frac{\lambda }{r}\right)}\right)\right)\frac{\psi_{1}^{*}}{1+r}-\frac{\lambda^{2}\sigma^{2}}{{\left(1+r\right)}^{2}}f\left(-\frac{\psi_{1}^{*}}{\left(1+r-\lambda \right)\left(\frac{\lambda }{r}\right)}\right) \end{array}\right) \end{array}$$

Therefore, the critical condition of IPO timing in *t* = 1 without the pricing constraint can be achieved.

If the pricing constraint exists, as shown in Equation (A8),

(A8) $$\begin{array}{c} Pr\left(NI_{i\neq j}^{1}\right)\left(\frac{P^{*}}{1+r}\right)+(1-\\ Pr\left(NI_{i\neq j}^{1}\right))\left(\begin{array}{c} Pr\left(I_{i\neq j}^{1}\right)E\left[payoff\;at\;t=2|\psi_{i},I_{i}^{2}\right]\\ +Pr\left(NI_{i\neq j}^{1}\right)E\left[payoff\;at\;t=3|\psi_{i},NI_{i}^{2}\right] \end{array}\right)={\left[G\left(\psi_{1}^{*}\right)\right]}^{N-1}\left(\frac{P^{*}}{1+r}\right)+\\ (1-\\ {\left[G\left(\psi_{1}^{*}\right)\right]}^{N-1})\left(\begin{array}{c} F\left(\;\frac{\left(1+r\right)\psi_{1}^{*}-P^{*}}{\lambda \left(1+r\right)}\right)E\left[payoff\;at\;t=2|\psi_{i},I_{i}^{2}\right]\\ +\left(1-F\left(\;\frac{\left(1+r\right)\psi_{1}^{*}-P^{*}}{\lambda \left(1+r\right)}\right)\right)E\left[payoff\;at\;t=3|\psi_{i},NI_{i}^{2}\right] \end{array}\right)=\\ {\left[G\left(\psi_{1}^{*}\right)\right]}^{N-1}\left(\frac{P^{*}}{1+r}\right)+(1-\\ {\left[G\left(\psi_{1}^{*}\right)\right]}^{N-1})\left(\begin{array}{c} Pr\left(\;\psi_{i}>\frac{P^{*}-\left(1+r\right)c_{1}}{\lambda \left(1+r\right)}\right)E\left[\frac{\psi_{i}+c_{2}}{1+r}|\psi_{i}>\frac{P^{*}-\left(1+r\right)c_{1}}{\lambda \left(1+r\right)}\right]\\ +Pr\left(\psi_{i}\leq \frac{P^{*}-\left(1+r\right)c_{1}}{\lambda \left(1+r\right)}\right)\frac{P^{*}}{{\left(1+r\right)}^{2}} \end{array}\right) \end{array}$$

and as shown in Equation (A9),

(A9) $$\begin{array}{l} E\left[\frac{\psi_{i}+c_{2}}{1+r}|\psi_{i}>\frac{P^{*}-\left(1+r\right)c_{1}}{\lambda \left(1+r\right)}\right]=E\left[\frac{\psi_{i}+c_{2}}{1+r}|c_{1}>\frac{P^{*}-\left(1+r\right)\psi_{i}}{\lambda \left(1+r\right)}\right]\\ \;\;\;\;\;\;\;\;\;\;\;\;\;\;\;\;\;\;\;\;=\frac{1}{F\left(\frac{\left(1+r\right)\psi_{i}-P^{*}}{\lambda \left(1+r\right)}\right)}\int_{\frac{P^{*}-\left(1+r\right)\psi_{i}}{\lambda \left(1+r\right)}}^{\infty }\frac{\psi_{i}+E\left[c_{2}|c_{1}\right]}{1+r}f\left(c_{1}\right)dc_{1}\\ \;\;\;\;\;\;\;\;\;\;\;\;\;\;\;\;\;\;\;\;=\frac{\psi_{i}}{1+r}+\frac{1}{1+r}\frac{1}{F\left(\frac{\left(1+r\right)\psi_{i}-P^{*}}{\lambda \left(1+r\right)}\right)}\int_{\frac{P^{*}-\left(1+r\right)\psi_{i}}{\lambda \left(1+r\right)}}^{\infty }\frac{E\left[c_{2}|c_{1}\right]}{1+r}f\left(c_{1}\right)dc_{1}\\ \;\;\;\;\;\;\;\;\;\;\;\;\;\;\;\;\;\;\;\;=\frac{\psi_{i}}{1+r}+\frac{1}{1+r}E\left[c_{2}|c_{1}>\frac{P^{*}-\left(1+r\right)\psi_{i}}{\lambda \left(1+r\right)}\right]\\ \;\;\;\;\;\;\;\;\;\;\;\;\;\;\;\;\;\;\;\;=\frac{\psi_{i}}{1+r}+\frac{1}{1+r}\left(0-\sigma^{2}\frac{-f\left(\frac{P^{*}-\left(1+r\right)\psi_{i}}{\lambda \left(1+r\right)}\right)}{1-F\left(\frac{P^{*}-\left(1+r\right)\psi_{i}}{\lambda \left(1+r\right)}\right)}\right)\\ \;\;\;\;\;\;\;\;\;\;\;\;\;\;\;\;\;\;\;\;=\frac{\psi_{i}}{1+r}+\frac{1}{1+r}\sigma^{2}\frac{f\left(\frac{P^{*}-\left(1+r\right)\psi_{i}}{\lambda \left(1+r\right)}\right)}{1-F\left(\frac{P^{*}-\left(1+r\right)\psi_{i}}{\lambda \left(1+r\right)}\right)} \end{array}$$

thus, the expected utility of enterprises in postponing the IPO at *t* = 1 is shown in Equation (A10).

(A10) $$\begin{array}{l} Pr\left(NI_{i\neq j}^{1}\right)\left(\frac{P^{*}}{1+r}\right)\\ +\left(1-Pr\left(NI_{i\neq j}^{1}\right)\right)\left(\begin{array}{c} Pr\left(I_{i\neq j}^{1}\right)E\left[payoff\;at\;t=2|\psi_{i},I_{i}^{2}\right]\\ +Pr\left(NI_{i\neq j}^{1}\right)E\left[payoff\;at\;t=3|\psi_{i},NI_{i}^{2}\right] \end{array}\right)={\left[G\left(\psi_{1}^{*}\right)\right]}^{N-1}\left(\frac{P^{*}}{1+r}\right)\\ +\left(1-{\left[G\left(\psi_{1}^{*}\right)\right]}^{N-1}\right)\left(\begin{array}{c} F\left(\;\frac{\left(1+r\right)\psi_{1}^{*}-P^{*}}{\lambda \left(1+r\right)}\right)\frac{\psi_{i}}{1+r}+\frac{1}{1+r}\sigma^{2}f\left(\frac{P^{*}-\left(1+r\right)\psi_{i}}{\lambda \left(1+r\right)}\right)\\ +\left(1-F\left(\;\frac{\left(1+r\right)\psi_{1}^{*}-P^{*}}{\lambda \left(1+r\right)}\right)\right)\frac{P^{*}}{{\left(1+r\right)}^{2}} \end{array}\right) \end{array}$$

## Appendix B. Derivation: The Real Option of IPO Timing

If $c_{2}<c_{2}^{*}\left(\psi_{i}\right)$, the strategic enterprises will choose to delay their IPO until *t* = 3, and we get Equation (A11).

(A11) $$\left(\psi_{i}\right)=Pr\left(c_{2}<c_{2}^{*}\left(\psi_{i}\right)\right)E\left[\frac{\psi_{i}+c_{3}}{{\left(1+r\right)}^{2}}\right]+\left(1-Pr\left(c_{2}<c_{2}^{*}\left(\psi_{i}\right)\right)\right)E\left[\frac{\psi_{i}+c_{2}}{1+r}\right]$$

The value of the IPO timing real option is shown in Equation (A12).

(A12) $$\begin{array}{ll} V_{2}\left(\psi_{i}\right)\equiv S\left(\psi_{i}\right) & -NS\left(\psi_{i}\right)\\ & =Pr\left(c_{2}<c_{2}^{*}\left(\psi_{i}\right)\right)E\left[\frac{\psi_{i}+c_{3}}{{\left(1+r\right)}^{2}}\right]+\left(1-Pr\left(c_{2}<c_{2}^{*}\left(\psi_{i}\right)\right)\right)E\left[\frac{\psi_{i}+c_{2}}{1+r}\right]\\ & -E\left[\frac{\psi_{i}+c_{2}}{1+r}\right]\\ & =Pr\left(c_{2}<c_{2}^{*}\left(\psi_{i}\right)\right)E\left[\frac{\psi_{i}+c_{3}}{{\left(1+r\right)}^{2}}\right]-Pr\left(c_{2}<c_{2}^{*}\left(\psi_{i}\right)\right)E\left[\frac{\psi_{i}+c_{2}}{1+r}\right]\\ & =Pr\left(c_{2}<c_{2}^{*}\left(\psi_{i}\right)\right)E\left[\frac{\psi_{i}+c_{3}}{{\left(1+r\right)}^{2}}-\frac{\psi_{i}+c_{2}}{1+r}|c_{2}<c_{2}^{*}\left(\psi_{i}\right)\right]\\ & =F\left(c_{2}^{*}\left(\psi_{i}\right)\right)\frac{1}{F\left(c_{2}^{*}\left(\psi_{i}\right)\right)}\int_{-\infty }^{c_{2}^{*}\left(\psi_{i}\right)}(\frac{\psi_{i}+E\left[c_{3}|c_{1}\right]}{{\left(1+r\right)}^{2}}\\ & -\frac{\psi_{i}+E\left[c_{2}|c_{1}\right]}{1+r})f\left(c_{1}\right)dc_{1}\\ & =\int_{-\infty }^{c_{2}^{*}\left(\psi_{i}\right)}\left(\frac{\psi_{i}+E\left[c_{3}|c_{1}\right]}{{\left(1+r\right)}^{2}}-\frac{\psi_{i}+E\left[c_{2}|c_{1}\right]}{1+r}\right)f\left(c_{1}\right)dc_{1} \end{array}$$

and as shown in Equations (A13) and (A14).

(A13) $$E\left[c_{2}|c_{1}\right]=\int_{-\infty }^{\infty }\left(\lambda c_{1}+\varepsilon_{2}\right)f\left(\varepsilon_{2}\right)d\varepsilon_{2}=\lambda c_{1}$$

(A14) $$E\left[c_{3}|c_{1}\right]=\int_{-\infty }^{\infty }\left(\lambda \left(\int_{-\infty }^{\infty }\left(\lambda c_{1}+\varepsilon_{2}\right)f\left(\varepsilon_{2}\right)d\varepsilon_{2}\right)+\varepsilon_{2}\right)f\left(\varepsilon_{2}\right)d\varepsilon_{2}=\lambda^{2}c_{1}$$

thus, we have Equation (A15).

(A15) $$V_{2}\left(\psi_{i}\right)=\int_{-\infty }^{c_{2}^{*}\left(\psi_{i}\right)}\left(\frac{\psi_{i}+\lambda^{2}c_{1}}{{\left(1+r\right)}^{2}}-\frac{\psi_{i}+\lambda c_{1}}{1+r}\right)f\left(c_{1}\right)dc_{1}$$

This derivation does not consider the pricing constraint, when the IPO is faced with the issue pricing constraint, the IPO timing real option is shown in Equation (A16).

(A16) $$\begin{array}{cc} V_{2}\left(\psi_{i}\right)\equiv S\left(\psi_{i}\right) & -NS\left(\psi_{i}\right)\\ & =Pr\left(c_{2}<c_{2}^{*}\left(\psi_{i}\right)\right)\frac{P^{*}}{{\left(1+r\right)}^{2}}+\left(1-Pr\left(c_{2}<c_{2}^{*}\left(\psi_{i}\right)\right)\right)E\left[\frac{\psi_{i}+c_{2}}{1+r}\right]\\ & -E\left[\frac{\psi_{i}+c_{2}}{1+r}\right]\\ & =Pr\left(c_{2}<c_{2}^{*}\left(\psi_{i}\right)\right)\frac{P^{*}}{{\left(1+r\right)}^{2}}-Pr\left(c_{2}<c_{2}^{*}\left(\psi_{i}\right)\right)E\left[\frac{\psi_{i}+c_{2}}{1+r}\right]\\ & =Pr\left(c_{2}<c_{2}^{*}\left(\psi_{i}\right)\right)E\left[\frac{P^{*}}{{\left(1+r\right)}^{2}}-\frac{\psi_{i}+c_{2}}{1+r}|c_{2}<c_{2}^{*}\left(\psi_{i}\right)\right]\\ & =F\left(c_{2}^{*}\left(\psi_{i}\right)\right)\frac{1}{F\left(c_{2}^{*}\left(\psi_{i}\right)\right)}\int_{-\infty }^{c_{2}^{*}\left(\psi_{i}\right)}\left(\frac{P^{*}}{{\left(1+r\right)}^{2}}-\frac{\psi_{i}+E\left[c_{2}|c_{1}\right]}{1+r}\right)f\left(c_{1}\right)dc_{1}\\ & =\int_{-\infty }^{c_{2}^{*}\left(\psi_{i}\right)}\left(\frac{P^{*}}{{\left(1+r\right)}^{2}}-\frac{\psi_{i}+\lambda c_{1}}{1+r}\right)f\left(c_{1}\right)dc_{1} \end{array}$$
